# Smiling won’t make you feel better, but it might make people like you more: Interpersonal and intrapersonal consequences of response-focused emotion regulation strategies

**DOI:** 10.1177/02654075221077233

**Published:** 2022-02-28

**Authors:** Nancy Bahl, Allison J Ouimet

**Affiliations:** 1 School of Psychology, 6363 University of Ottawa, Ottawa, ON, Canada

**Keywords:** Emotion regulation, expressive dissonance, expressive suppression, psychophysiology, memory, interpersonal relationships, experiment

## Abstract

Emotion regulation (ER) is integral to well-being and relationship quality. Experimental studies tend to explore the intrapersonal effects of ER (i.e. impacts of ER on oneself) and leave out the interpersonal impacts (i.e. the bidirectional impact of ER on the regulator and partner). The ER strategy expressive suppression shows maladaptive interpersonal and intrapersonal consequences during distressing conversations. We aimed to explore whether other ER strategies that modify facial expressions (i.e. expressive dissonance) have similar consequences to suppressing emotional expressions. We randomly assigned 164 women participants to use expressive dissonance and expressive suppression or to naturally express emotions, while engaging in a conversation task with a confederate. We observed intrapersonal outcomes, including electrodermal activity and self-reported affect throughout the experiment, and memory performance after. Video coders unaware of the study goals assessed the conversation on interpersonal qualities (e.g. friendliness and likeability). There were no differences between conditions on intrapersonal outcomes. Participants engaging in expressive dissonance, however, were rated more positively, and participants in the expressive suppression condition were rated more negatively on interpersonal qualities, relative to the control condition. Although neither strategy appeared to impact the participant, intrapersonally, both notably influenced the observer’s impression of the participant.

Emotion regulation (ER) involves using strategies to modulate emotional expressions to meet individual goals or to abide by social norms (e.g. showing happiness when receiving an undesired gift; Gross, 1998). Emotion regulation strategies can be classified as antecedent versus response-focused ER strategies (Gross, 1998). Antecedent-focused ER strategies are used before an emotion is evoked. Cognitive reappraisal, for example, is an antecedent-focused emotion regulation strategy, which people use to alter their interpretation of a situation and modify its emotional impacts (e.g. concluding that your friend did not phone you back because they were sleeping). People use response-focused emotion regulation strategies, in contrast, once an emotion is already underway, with the goal of altering an emotional output. Expressive suppression is the most widely studied response-focused ER strategy and involves inhibiting outward facial expressions following the start of an affective experience (i.e. displaying a poker or neutral facial expression; Gross, 1998).

Researchers use experimental designs to measure the impacts of ER strategies, by manipulating the emotion regulation strategy that participants are asked to engage in when participating in an emotion-eliciting task (e.g. picture-watching or speech task). Some researchers have found that using expressive suppression is less helpful than other ER strategies in terms of intrapersonal outcomes (i.e. effects on oneself); participants who suppress their expressions tend to experience an increase in sympathetic nervous system arousal, difficulties in memory accuracy and decreased self-reported positive mood (see Webb et al., 2012). Moreover, people with mood and anxiety disorders tend to use suppression inflexibly and more often than people without those clinical diagnoses (Aldao et al., 2010), which highlights the potential long-term disadvantages of frequently using this strategy.

In most experimental studies on ER, researchers measure intrapersonal outcomes of ER strategies – the impact on oneself – and pay little attention to the interpersonal outcomes of ER strategies – the impact on others, or on relational dynamics. The process of interpersonal ER refers to *intentionally* attempting to alter someone *else’s* emotional experience or intentionally engaging someone else in altering our emotional experience (e.g. Niven, 2017; Zaki & Williams, 2013). However, people’s attempts to regulate their own emotions (rather than the emotions of others) can also have unintentional social impacts. How do different ER strategies help or hinder our relationships? How do people perceive us? The quality of our social interactions may be affected by the emotion regulation strategies that we use (Gross, 1998).

The strategies that people use to regulate their own emotions can impact the quality of their interpersonal interactions and relationships (Gross & John, 2003). In one meta-analysis of research focused on emotional expression or suppression and social variables, Chervonsky and Hunt (2017) found that emotional suppression of any emotion was associated with poorer overall social well-being, including social liking by unacquainted peers. Moreover, these associations were stronger for women participants than for men, suggesting that the social cost of emotional suppression may be higher for women. However, the vast majority of studies included in the meta-analysis were correlational or longitudinal and relied on participants’ self-report. As such, it is unclear whether ER strategy use causes specific social outcomes, and whether those outcomes, as perceived by the self, are similar to those perceived by other social actors. Experimental designs with behavioural outcomes are vital to understanding the impact of different ER strategies on social outcomes.

Although limited, there are a few experimental studies in which researchers examined the physiological, cognitive, affective, and social consequences of expressive suppression during social interactions of unacquainted pairs. Butler et al. (2003) recruited unacquainted pairs of women to discuss an upsetting topic; one partner was given instructions on the strategy to use to regulate their emotions. Those who suppressed their expressions reported feeling more negative emotions during the conversation, relative to those who reappraised or received no instructions. Crucially, suppression was shown to increase blood pressure in *both* the regulators and their partners, relative to the other two conditions. The partners of suppressors reported less rapport and willingness to form a friendship. Peters et al. (2014) asked participants (men and women) to use either suppression or exaggeration while discussing a negative emotion-eliciting video with an unacquainted opposite-sex partner. The partners reported that suppressors were poor communicators and expressed fewer facial expressions, emotions and gestures. Additionally, both participants of the suppression dyads exhibited increased sympathetic nervous system arousal during the conversation task. Finally, Butler et al. (2008; Studies 1 and 2) found that participants reported being more likely to help a person (in a vignette, Study 1; a confederate, Study 2) who expressed feeling a negative emotion compared to people who experienced, but did not express a negative emotion.

Memory accuracy is also negatively impacted by expressive suppression. Richards and Gross (2005) found that people who suppressed their expressions while discussing a relationship conflict with their partner remembered less of the discussion, relative to those who reappraised or received no specific instructions to regulate. The authors attributed these findings to the constant self-monitoring of one’s own facial displays, which may distract the regulator from his/her partner, reducing their ability to retain or remember pieces of the conversation (Richards & Gross, 2005).

Taken together, the findings from experimental research suggest that suppression has the potential to significantly undermine social functioning and relationship quality, via its effects on communication, psychophysiology, cognition, and affect, among both conversation participants. Because expressive suppression is a response-focused strategy that may be associated with social consequences, it is crucial to examine whether other response-focused ER strategies (i.e. strategies that modulate facial expressions) lead to similar impacts. Indeed, researchers have typically categorized response-focused strategies as maladaptive (e.g. Gross, 1998), in that they counterproductively up-regulate, rather than down-regulate emotional responses. However, there are social scenarios in which suppressing our emotional expression may actually be socially beneficial (e.g. suppressing an anger expression in a business meeting). Thus, we need to better understand social contexts in which it is helpful to regulate (either by suppression or dissonance) evoked emotions. In a recent review, English and Eldesouky (2020) highlighted the need for research extending our understanding of how strategies other than expressive suppression lead to better or worse social outcomes.

Expressive dissonance occurs by displaying the opposite of how one feels (e.g. smiling when feeling sad). Expressive dissonance represents an incongruency between the emotion felt and the emotion presented. To our knowledge, only three studies have explored the intrapersonal impacts of expressive dissonance, specifically, smiling during a stressful task. Robinson and Demaree (2007) and Pelt et al. (2018) found that relative to a control condition, participants who engaged in expressive dissonance (i.e. smiling) while watching negative film-clips displayed greater sympathetic nervous system arousal and performed poorly on a memory task assessing social and visual information presented in the film-clips. In our more recent study (Bahl & Ouimet, 2022), we recruited a relatively large sample of undergraduate women and measured the intrapersonal impacts of expressive dissonance versus expressive suppression and a control condition. However, we were unable to replicate the previous findings. Women who used expressive suppression or expressive dissonance did not differ from each other or from a control condition on intrapersonal outcomes (i.e. psychophysiology, memory accuracy and self-reported affect), while viewing negative emotion-evoking images.

Given that this finding is contrary to the literature on response-focused emotion regulation, and is one of only three studies looking specifically at the intrapersonal impacts of expressive dissonance, our conclusions on the potential helpfulness of expressive dissonance may be premature. Indeed, in the clinical literature, expressive dissonance, particularly smiling when distressed, has been framed as a helpful distress-tolerance skill. The half-smile technique (i.e. gentle upturn of the lips) is a distress-tolerance skill, developed from research that feedback from facial expressions influences emotions and emotion-related behaviours (Linehan, 1993).

It is therefore possible that expressive dissonance may be more helpful in regulating emotions (e.g. decreased psychophysiology and improved self-reported affect) relative to expressive suppression. Additionally, to our knowledge, no studies have explored the interpersonal impacts of using expressive dissonance. This gap is unfortunate, given that emotion regulation strategies have implications not only for individuals but also for the development and maintenance of relationships with others (e.g. English & Eldesouky, 2020).

In the current study, we investigated whether expressive dissonance leads to more positive intrapersonal and interpersonal outcomes than expressive suppression, given the documented benefits of expressing positive emotions during social interactions (Chervonsky & Hunt, 2017). Participants self-identifying as women participated in an unstructured ‘getting to know you’ task with an unacquainted male confederate. The participants were randomized and given specific instructions to engage in expressive suppression (i.e. show no expression), expressive dissonance (i.e. smile throughout the task) or control condition (i.e. express naturally); they then engaged in an unstructured ‘getting to know you’ task with an unacquainted male confederate using those instructions. Our main outcomes included repeated measures of electrodermal activity (EDA) and self-reported affect, and three memory tasks (free recall, cued recall and recognition) administered after the task.

We registered our hypotheses, methods and analysis plan on the Open Science Framework (OSF) before data collection began (https://doi.org/10.17605/OSF.IO/YU94E) and uploaded our original de-identified dataset^1^ (i.e. before any data cleaning) to OSF. We hypothesized that participants in the Expressive Suppression condition would show greater sympathetic nervous system arousal (H1), reduced positive mood (H2), and increased peak anxiety (H3) during the conversation task, relative to those in the Control condition, who in turn, would show greater sympathetic nervous system arousal, reduced positive mood, and increased peak anxiety relative to those in the Expressive Dissonance condition. We also hypothesized that participants in the experimental conditions would perform worse on memory tasks (i.e. free recall, cued recall, and recognition task), relative to the Control condition (H4). Finally, we hypothesized the judges, unaware of the study goals, design or hypotheses, would rate people in the Expressive Dissonance condition higher and the Expressive Suppression condition lower on interpersonal qualities (e.g. friendliness), relative to the control condition (H5).^2^

## Method

### Participants

All methods and procedures were approved by the Research Ethics Board at the University of Ottawa (Ontario, Canada) prior to beginning data collection. We recruited 164 women^3^ participants (*M*_age_ = 18.68 years, *SD* = 1.96, range 17–32 years) via the participant pool at the university. Participants were required to be fluent in English to participate in the tasks. All participants were residing in Canada during the time of the experiment. Participants identified themselves as^4^ White (59.2%), Black (6.8%), Latinx (4.1%), Asian (12.2%), South Asian (10.2%), Indigenous (4.1%), Middle Eastern (9.5%) Caribbean (3.4%), European (10.9%), Other (4.1%), and preferred not to answer (.70%). All participants identified as women. In regard to sexual orientation, participants described themselves as heterosexual (86.7%), lesbian (1.2%), bisexual (5.4%), bicurious (3.0%), unsure (1.2%), and preferred not to answer (.6%). The majority (95.2%) of participants were full-time students (i.e. 12 credits or more per semester), whereas 3.0% were part-time students. Most participants (59.0%) were not currently employed, about 37.3% were employed part-time and 1.8% were employed full time (i.e. more than 25 hours of paid work per week). Participants reported a household income of more than $80 000 CAD (44.6%), $60 000–$80 000 CAD (13.3%), $40 000–$60 000 CAD (14.5%), $20 000–$40 000 CAD (7.8%) or less than $20 000 CAD (3.6%). Approximately 8% of participants reported a past diagnosis of a mood or anxiety disorder. We did not ask participants whether they lived with other disabilities. Participants were compensated with one course credit.

### Experimental manipulation

Participants were randomly assigned to one of three conditions: expressive dissonance, expressive suppression, or control. Participants were then given instructions on the conversation task:

“I would like you to take part in a conversation with another participant who is waiting in the other experiment room. Your task is to try to acquaint yourself with this person, similar to what you may do when meeting someone for the first time. The conversation will last for at least 10 minutes, and you will be recorded using a video camera. During the conversation, a judge, who will be watching you two from the other room, through cameras (point at the camera on the tripod) will be evaluating you on the following criteria: clarity, interest, content, poise, style, and management of emotions. The conversation will be video recorded so that a group of researchers can also rate your conversation on the criteria mentioned at a later date.”

Depending on the condition, participants were given the following instructions (underlined phrases indicate manipulated portions of instructions):

### Expressive Suppression condition (*n* = 54)

“This task may produce a range of negative feelings, like anxiety. People tend to deal with these feelings in different ways. For the purposes of this study, it’s extremely important that you deal with these feelings by not showing any of your emotions during the conversation. That is, please maintain a straight face so that I and the other participant will not be able to see that you are feeling anything at all. Remember, do not show your emotions at any time during this task.”;

### Expressive Dissonance condition (*n* = 54)

“This task may produce a range of negative feelings, like anxiety. People tend to deal with these feelings in different ways. For the purposes of this study, it’s extremely important that you deal with these feelings by showing happiness during the conversation. That is, please maintain a natural smile with teeth so that I and the other participant will be able to see that you are feeling happy. Remember, smile as often as you can during this task”;

### Control condition (*n* = 54)

“This task may produce a range of negative feelings, like anxiety. People tend to deal with these feelings in different ways. For the purposes of this study, it’s extremely important that you deal with these feelings by showing your feelings in any manner that you wish during the conversation. That is, please behave in such a way that I and the other participant might or might not be able to see what you are feeling. Remember, show your emotions if you wish at any time during this task.”

### Conversation and memory task

#### Conversation task

Participants self-identifying as women participated in an unstructured ‘getting to know you’ task with an unacquainted male confederate. We selected a conversation task as the social-evaluative task, given its documented effectiveness creating stress and enabling observation of interpersonal outcomes within a laboratory setting (e.g. Voncken & Bögels, 2008). The conversation confederate partners were men, given evidence that opposite-gender dyads create more objective and subjective levels of anxiety than same-gender dyads (Mendes et al., 2003). To contribute to a distressing situation, all confederates were trained to act more ‘aloof’ with minimal emotional expression and to leave the onus of the conversation to the participant. During silences in the conversation, confederates were instructed to wait approximately 7 seconds before asking a question to the participant, to allow the participant to fill in the silence. Confederates were also trained to provide five specific pieces of information (name, university program, career aspiration, birth month, and favourite TV show) throughout the conversation, which formed the basis of the memory tasks later (of which the participants were unaware).

To ensure that participants understood the regulation instructions assigned, they were asked to partake in up to three practice rounds by responding to a question from the experimenter (e.g. ‘What is your favourite season and why?’), while engaging in the strategy assigned. The experimenter was thus able to verify that participants maintained a neutral expression (ES condition), smiled (ED condition), or expressed emotions naturally (control condition) or provide feedback until they were able to do so, prior to engaging in the conversation task. The videos were coded for affective expressions (i.e. positive, negative, and neutral expressions) to examine whether participants adhered to the emotion regulation strategy assigned (see the Data Preparation section).

#### Memory task

Participants completed three memory tasks: free recall, cued recall, and a multiple-choice recognition task, for information relayed by the confederate during the conversation. In the free recall task, participants wrote down as many pieces of information about the confederate as they could recall from the conversation. In the cued recall task, participants answered five open-ended questions of information presented by the confederate (i.e. ‘What was his name; University program; career aspiration; favourite TV show; birth month’). In the multiple-choice recognition task, participants answered five multiple choice questions identical to what was asked in the cued recall task.

### Measures

#### Baseline Questionnaires

Because Aldao et al. (2010) suggest that people with mood and/or anxiety disorders use expressive suppression more than people without, we included measures of social anxiety (Social Phobia Inventory: SPIN; Connor et al., 2000) and depression (Depression Anxiety Stress Scale-21 – Depression Subscale: DASS-DEP; Lovibond & Lovibond, 1995) symptomatology as covariates in a separate set of our main analyses. We also included measures of ER, to account for potential condition differences in 1) difficulties with ER (Difficulties in Emotion Regulation Scale: DERS; Gratz & Roemer, 2004) and 2) degree of specific emotion regulation strategy use (Emotion Regulation Questionnaire: ERQ; Gross & John, 2003).

*Depression Anxiety Stress Scale – Depression Subscale* (DASS-DEP; Lovibond & Lovibond, 1995): The depression subscale of the DASS is a 7-item self-report questionnaire, which measures depression symptomatology (e.g. ‘I felt that I had nothing to look forward to’), on a 4-point Likert scale (0 = ‘Did not apply to me at all’, 3 = ‘Applied to me very much’ or ‘most of the time’). The DASS-DEP subscale demonstrates excellent internal consistency (α = .92; Antony et al., 1998).

*Social Phobia Inventory* (SPIN; Connor et al., 2000): The SPIN is a 17-item self-report questionnaire, which assesses social anxiety symptoms experienced over the past week, on a 5-point Likert scale (0 = ‘Not at all’, 4 = ‘Extremely’). The SPIN comprises three subscales: fear (e.g. ‘I am afraid of people in authority’; six items), avoidance, (‘I avoid talking to people I don’t know’; seven items,) and physiological arousal (‘Heart palpitations bother me when I am around people’; four items). The SPIN demonstrated good convergent validity, discriminant validity, and test–retest reliability (.78 ≤ *r* ≤ .89), and good to excellent internal consistency in people with social anxiety disorder (.87 ≤ *α* ≤ .94) and healthy participants (.82 ≤ *α* ≤ .90; Connor et al., 2000).

*Difficulties in Emotion Regulation Scale* (DERS; Gratz & Roemer, 2004): The DERS is a 36-item self-report questionnaire that measures the degree of emotion dysregulation. The DERS includes six subscales: Non-acceptance of emotional responses (e.g. ‘When I’m upset, I become angry with myself for feeling that way’; six items), Difficulties engaging in goal-directed behaviour (e.g. ‘When I’m upset, I have difficulty focusing on other things’; five items), Impulse control difficulties (e.g. ‘When I’m upset, I lose control over my behaviours’; six items), Lack of emotional awareness (e.g. ‘I care about what I am feeling’ [reverse-scored]; six items), Limited access to emotion regulation strategies (e.g. ‘When I’m upset, it takes me a long time to feel better’; eight items), and Lack of emotional clarity (e.g. ‘I am confused about how I feel’; five items). The items are on a 5-point Likert scale (1 = ‘Almost never’, 0%–10%, 5 = ‘Strongly agree’, 91–100%). The individual subscales and total scale score demonstrated good to excellent internal consistency (.80 ≤ α ≤ .93), and the DERS has documented adequate construct and predictive validity (Gratz & Roemer, 2004).

*Emotion Regulation Questionnaire* (ERQ; Gross & John, 2003): The ERQ measures people’s use of two emotion regulation strategies: Cognitive Reappraisal (ERQ-REA; e.g. ‘When I want to feel more positive emotion (such as joy or amusement), I change what I’m thinking about’; four items) and Expressive Suppression (ERQ-SUPP; ‘When I am feeling negative emotions, I make sure not to express them’; six items). The 10-item self-report questionnaire includes an 8-point Likert scale (0 = ‘Strongly disagree’, 7 = ‘Strongly agree’). The ERQ demonstrated good convergent and discriminant validity; the reappraisal (*α* = .82) and suppression (*α* = .76) subscales demonstrated good and acceptable internal consistency, respectively (Gross & John, 2003).

To measure the degree to which people engage in expressive dissonance, we added three items to the ERQ questionnaire (ERQ-DIS) in a previous study (Bahl & Ouimet, 2022): ‘When I want to feel less negative emotion (such as sadness or anger), I try to smile in order to feel happier’; ‘I control my emotions by expressing the opposite of how I am really feeling (e.g. showing happiness when feeling sad)’; ‘When I want to feel more positive emotion (such as joy or amusement), I try to smile in order to feel happier’. The ERQ-DIS subscale showed good internal consistency (*α* = .82; Bahl & Ouimet, 2022).

#### Manipulation Check Questionnaire

After the conversation task, participants completed a 5-item questionnaire (see Supplemental Materials: Supplementary Appendix C), which assessed the degree to which they believed they engaged in the ER strategy assigned during the conversation task. If participants in the experimental conditions reported not consistently engaging in the strategy assigned (i.e. a score of 0 = ‘Not at all’, 1 = ‘Rarely’ or 2 = ‘Somewhat’), their data were considered for exclusion.

#### Outcome measures

To measure self-reported affect, we included (1) the Emotion Experience Scale (see Butler et al., 2003) to assess positive and negative emotions throughout the experiment (before baseline, anticipation, conversation task, and recovery task) and (2) the Subjective Units of Distress Scale (SUDS; Wolpe, 1958) to measure state distress before the conversation task, but after instructions were given (i.e. anticipatory anxiety), and immediately afterwards, but related to most anxiety felt during the conversation task (i.e. peak anxiety). To measure sympathetic nervous system activation, we recorded electrodermal activity (EDA) throughout the study. To assess for effects of condition on memory, we included a memory task questionnaire.

*Emotion Experience Scale:* The emotion experience (EE) scale is a 16-item questionnaire, used in Butler et al. (2003) to assess eight negative emotions (negative emotion subscale: anger, anxiety, fear, disgust, sadness, frustration, guilt, and shame) and eight positive emotions (positive emotion subscale: amusement, happiness, joy, love, interest, excitement, pride, and pleasantness). The questionnaire is rated on an 11-point Likert scale. The Negative (*α* = .87) and Positive (*α* = .78) Emotion scales demonstrated good internal consistency (Butler et al., 2003).

*Subjective Units of Distress Scale* (SUDS; Wolpe, 1958): The SUDS measures state distress. It is widely used in behavioural research and therapy to measure feelings of anxiety (e.g. Milosevic & Radomsky, 2008). Respondents rated the extent to which they felt distressed on a scale from 0 (‘totally relaxed’) to 100 (‘highest anxiety/distress that you have ever felt’) by drawing a line through a visual thermometer. The SUDS correlates with physiological measures of stress (Thyer et al., 1984).

*Electrodermal Activity*: EDA measures electrical conductance from sweating (an indicator of sympathetic nervous system arousal; Roy et al., 2012). We collected EDA via a portable ambulatory device. Two electrodes were placed on the thenar and hypothenar muscles on the non-dominant palm. The electrodes were Biopac TSD203 transducers filled with Biopac Skin Conductance Electrode Paste. Data were amplified using Biopac’s GSR100C amplifier using a gain of 10 Hz and a low-pass filter of 10 Hz.

### Procedure

The experiment took place in the INSPIRE laboratory, a well-equipped shared psychophysiology facility at the University of Ottawa. Participants were informed that the aim of the study was to better understand the relationship between emotions and likeability during a behavioural task. After the consent process, participants completed the EES, a sociodemographic measure, and baseline self-report questionnaires (DASS, SPIN, ERQ and DERS). The experimenter placed two EDA electrodes onto the participant’s hand, and participants completed a 3-minute rest (baseline) period. Participants then received instructions on the conversation task, including the experimental manipulation of emotion regulation instructions (random assignment).

Participants completed the second EES and the anticipatory SUDS measure. After the practice round(s), the experimenter brought the confederate into the room. The confederate also had electrodes on their palm, to provide evidence that they were ‘another participant’. The experimenter explained that the task was about to begin and that they could stop the task at any time by holding up a ‘Stop’ sign, or participate up to 10 minutes. The experimenter then set the camera and tripod in front of the participant and confederate. Participants were told that the conversation task was recorded and were falsely informed that the videos would be rated on indicators of anxiety (e.g. rate of speech and eye contact) by external judges. The experimenter then began recording EDA and turned the camera on. Once the pair said ‘stop’ or when 10 minutes were up, the experimenter returned and escorted the confederate out of the room. Participants completed the third EES and the peak anxiety SUDS, prior to completing a 3-minute rest (recovery) period. Participants completed the last EES and then the three memory tasks (one at a time). Finally, participants completed the manipulation check questionnaire to assess reported adherence to their condition instructions.

During debriefing, participants completed an additional consent form to include their data, after learning about the deception. All participants consented to include their data. The duration of the experiment was approximately 60 minutes.

### Data preparation

#### Electrodermal activity

We computed EDA in 30-second intervals during the conversation task and 10-second intervals during the baseline and recovery periods. We took the mean of each interval and then calculated the average of all intervals.

### Video coding

#### Emotional expression

Five women undergraduate volunteers (unaware of the study goals, design or hypotheses) were asked to code the conversation task videos for emotion-expressive behaviours, specifically affective expressions (i.e. positive, negative, and neutral expressions). Volunteers coded facial expressions during utterances (i.e. statements until a turn of speech – either the confederate jumped in or the affect changed) and sorted them into Positive Affect (i.e. displays of enjoyment, smiling), Neutral (i.e. neutral facial expression), and Negative Affect (comprising internalizing negative affect – displays of fear/anxiety, and externalizing negative affect – displays of frustration/aggression/anger). Because conversation length varied between participants, we used proportions to assess frequency of types of facial expressions and unresponsiveness (i.e. divided the number of positive, negative, or neutral expressions by the total number of expressions and divided the number of seconds of responsiveness by the length of the conversation). Video coders were also asked to record the number of times participants were non-responsive (i.e. when the confederate finished an utterance and the participant did not respond in 2 seconds).

#### Interpersonal qualities

The five undergraduate volunteers who coded the conversation task for expressions also coded the videos for social interaction qualities using the *Reysen Likeability Scale* (Reysen, 2005). Research assistants rated the participants on friendliness, likeability, warmth and approachability on a 7-point Likert scale (‘Very strongly disagree’ = 0 to ‘Very strongly agree’ = 6). We added an additional characteristic ‘easy to get along with’ – making it five characteristics.

We calculated interrater reliability for variables measured through video coding using intraclass correlation coefficient (ICC) for 10% of our videos. The ICC ranged from .19 (poor reliability for determining negative expressions) to .78 (good reliability for coding positive expressions). Given the poor reliability of negative expressions, we excluded that variable from our analyses. We found excellent reliability for unresponsiveness (.97). Across the interpersonal qualities, the overall ICC was 0.82 and ranged from .75 to .97 (see Table 1).

**Table 1.** Descriptive data for outcome variables.

	Expressive suppression					Expressive dissonance					Control				
	EDA	EEPOS	EENEG	SUDS 1	SUDS 2	EDA	EEPOS	EENEG	SUDS 1	SUDS 2	EDA	EEPOS	EENEG	SUDS 1	SUDS 2
*Mean* (*SD*)															
Baseline	.41 (.31)	35.25 (16.78)	13.77 (9.86)			.43 (.63)	35.07 (16.55)	15.15 (10.98)			.43 (.36)	33.59 (14.69)	17.61 (14.37)		
Anticipation		27.62 (16.79)	17.11 (11.98)				28.21 (18.64)	18.48 (10.29)				27.68 (16.49)	18.00 (11.67)		
Conversation	.53 (.32)	21.02 (15.34)	17.74 (10.81)	38.26 (21.67)	42.49 (22.12)	.52 (.32)	25.02 (16.98)	19.52 (12.13)	38.98 (16.09)	38.36 (20.72)	.52 (.35)	22.45 (16.05)	19.07 (12.54)	38.57 (22.31)	40.32 (23.50)
Recovery	.52 (.42)	23.41 (16.89)	9.15 (7.60)			.43 (.34)	24.29 (16.76)	10.38 (9.25)			.55 (.49)	20.96 (14.88)	12.35 (12.01)		

*Note:* EDA = electrodermal activity, EEPOS = emotional experience positive subscale; EENEG = emotional experience, negative subscale, SUDS 1 = anticipatory anxiety, SUDS 2 = peak anxiety.

#### Memory task coding

The memory task was coded by five other raters (three men and two women volunteers) unaware of the study goals, design or hypotheses. While watching the conversation task videos, volunteers coded the memory task questionnaires for number of hits (i.e. number of correctly identified statements), number of misses (i.e. number of incorrectly added statements), and the recall of standardized information (i.e. the five statements presented by the confederate to everyone).

We calculated an interrater reliability score (using ICC) for the number of hits, misses, and standardized information, using 10% of our videos. The agreement between hits (.93) and standardized information (.96) showed excellent reliability. The ICC for misses (.72) had good reliability.

### Manipulation Check

Similar to Bahl and Ouimet (2022), we took a stepped approach to including only participants who engaged in the appropriate manipulated ER strategy (see percentage breakdowns for each item in Supplemental Materials: Supplementary Appendix A, Table A1). Thirty-three participants (Suppression condition: 26; Dissonance condition; 7) reported in the manipulation check questionnaire that they did not engage adequately in the strategy assigned during the conversation task; thus, we considered their data for exclusion using the video coding of emotional expressions. We looked at the proportion of positive or neutral facial expressions that was determined for each of the 33 participants. Ultimately, we excluded data from two participants in the suppression and five participants in the dissonance condition as their proportions of neutral and positive expressions, respectively, were under 0.5.

During debriefing, we asked each participant whether they had any suspicions about the conversation partner. We excluded data from two participants who reported knowing their conversation partner was a confederate and another eight participants who withdrew from the experiment after learning about the conversation task. The final sample size was 147 (ES *n* = 53, ED *n* = 47, Control *n* = 47).

### Analysis Plan

To examine how ER strategies affect psychophysiology (H1), affect (H2 and H3), memory accuracy (H4), and interpersonal qualities (H5), during a conversation task, we used an experimental mixed-measures design, with Condition (Expressive Suppression, Expressive Dissonance and Control) as the between-participants factor and Time (Baseline, Conversation task and Recovery), as the within-participants factor. We used Least Square Difference (LSD) for pairwise planned comparisons across all analyses.

To measure the impact of condition on sympathetic nervous system arousal (H1: EDA), we conducted a 3 (condition) × 3 (time: baseline, conversation task, and recovery) repeated measures ANOVA. To measure the impact of condition on positive and negative emotion (H2: EE subscales, respectively), we conducted two 3 (condition) × 4 (time: before baseline, anticipation, conversation task, and recovery task) repeated measures ANOVAs. We conducted a second set of exploratory analyses to measure the effect of condition on psychophysiological arousal and affect, controlling for social anxiety and depression symptoms. To measure the impact of condition on self-reported anxiety (H3: SUDS), we conducted a 3 (condition) × 2 time (anticipation and peak anxiety) repeated measures ANOVA.

To measure the impact of condition on memory (H4), we conducted five one-way ANOVAs with (1) hits (free recall task), (2) misses (free recall task), (3) standardized information (free recall task), (4), cued recall, and (5) recognition memory as the dependent variables, respectively.

We conducted eight one-way ANOVAs to examine the differences in proportions of positive and neutral facial expressions, unresponsiveness, and differences in interpersonal qualities (H5) between conditions.

## Results

### Descriptive statistics and baseline differences

We assessed for normality for our self-reported measures; the values for skewness (−.49 to 2.0) and kurtosis (−0.85 to 6.6) across self-reported measures did not violate the assumption of normality (i.e. values did not exceed +/− 3 skewness and +/− 10 kurtosis; Kline, 2008). No participants (whose data were included in our analyses) had missing data in their self-reported questionnaires. Participants across conditions did not differ on age or scores on the baseline measures (see Table 2). Additionally, there were no differences between conditions on the number of participants who reported a past diagnosis of a mood or anxiety disorder (*χ2* = 4.2, *p* = .38).

**Table 2.** Descriptive data, internal consistencies and between condition differences for baseline measures.

Variables	Expressive suppression (*mean*, *SD*)	Expressive dissonance (*mean*, *SD*)	Control (*mean*, *SD*)	*Total* (*mean*, *SD*)	α	*F*	*p*
Age	18.83 (1.95)	18.37 (1.40)	18.85 (2.51)	18.7 (2.01)	—	.87	.42
DASS-DEP	4.85 (4.86)	5.59 (5.50)	5.74 (4.57)	5.37 (4.97)	.91	.46	.63
DERSTOT	97.51 (15.17)	96.83 (18.39)	98.93 (17.40)	97.7 (16.86)	.85	.19	.83
SPIN	24.25 (12.89)	27.61 (12.36)	27.66 (15.19)	26.4 (13.52)	.92	1.06	.35
ERQSUP	13.43 (4.84)	14.11 (5.04)	13.75 (5.41)	13.75 (5.06)	.77	.22	.81
ERQ-REA	30.30 (5.88)	29.33 (6.26)	28.04 (4.72)	29.27 (5.70)	.83	1.99	.14
ERQ-DIS	17.91 (6.16)	20.46 (6.15)	18.37 (5.27)	18.86 (5.95)	.77	2.56	.08
EDA1	.41 (.31)	.43 (.63)	.43 (.36)	.42 (.45)		.027	.97
EEPOS1	35.25 (16.78)	35.06(16.55)	33.59 (14.69)	34.66 (15.97)	.79	.154	.86
EENEG1	13.77 (9.86)	15.15(10.98)	17.61 (14.37)	15.44 (11.84)	.90	1.33	.27

*Note:* DASS-DEP = Depression Anxiety Stress Scale, Depression Subscale, DERSTOT = Difficulties in Emotion Regulation, Total Scale; SPIN = Social Phobia Inventory; ERQSUP = Emotion Regulation Questionnaire, Suppression Subscale, ERQ-REA = Emotion Regulation Questionnaire, Reappraisal Subscale, ERQ-DIS = Emotion Regulation Questionnaire, Dissonance Subscale, EDA1 = electrodermal activity, baseline EEPOS1 = emotional experience positive subscale at baseline; EENEG1 = emotional experience, negative subscale at baseline.

**Table 3.** Outcome and descriptive data for the recall and recognition memory task and facial expression video coding.

Condition	Expressive dissonance			Expressive suppression			Control			
	*Mean (SD)*	Min	Max	*Mean (SD)*	Min	Max	*Mean (SD)*	Min	Max	ICC
*Memory Task*										
Hits	10.96 (4.05)	2	25	12.0 (4.15)	0	20	10.59 (4.61)	0	21	.93
Misses	.62 (.83)	0	3	.82 (1.09)	0	5	.61 (.86)	0	3	.72
Standard information	2.87 (1.06)	0	5	2.86 (1.15)	0	4	2.91 (1.15)	0	5	.96
Cued recall	3.59 (.85)	2	5	3.47 (1.1)	0	5	3.66 (1.07)	2	5	
Recognition	4.32 (.73)	3	5	4.28 (.97)	1	5	4.34 (.82)	2	5	
*Facial Expressions*										
Positive	.46(.23)	0	1	.12 (.17)	0	.62	.30(.19)	.02	.73	.78
Neutral	.50(.21)	0	.93	.85(.17)	.38	1	.64(.19)	.28	.98	.72
Negative	.04(.05)	0	.21	.03(.06)	0	.26	.05(.07)	0	.26	.19
Responsiveness	15.85	0	132	20.92	0	95	19.78	0	73	N/A
*Interpersonal Qualities*										
Friendly	4.96 (.79)	3	6	3.49 (.83)	2	5	4.41 (.91)	2	6	.97
Likeable	4.89 (.88)	3	6	3.92 (.89)	2	6	4.33 (.94)	3	6	.95
Warm	4.28 (1.15)	1	6	2.84 (.90)	1	5	3.76 (1.12)	1	6	.75
Approachable	4.59 (1.09)	2	6	3.47 (1.06)	1	5	4.11 (1.04)	2	6	.86
Easy-going	4.65 (1.06)	2	6	3.94 (1.05)	2	6.00	4.39 (1.08)	2	6	.77

### Intrapersonal consequences

Due to violation of sphericity assumption, we used the Huynh-Feldt correction (EDA: χ^2^(2) = 10.89, *p* = .004; EE positive subscale: χ^2^(5) = 57.49, *p* = .000; EE negative subscale: χ^2^(5) = 45.29, *p* = .000). There was no condition by time interaction for EDA (*F*_3.8,263.50_ = .79, *p* = .53, *η*^2^ = .01), EE positive subscale (*F*_4.81,344.34_ = 1.05, *p* = .39, *η*^2^ = .01), or EE negative subscale (*F*_5.08,197.27_ = .65, *p* = .67, *η*^2^ = .009). There were no main effects of condition (*F*_2,138_ = .12, *p* = .89, *η*^2^ = .002) on EDA, positive affect (*F*_2,143_ = .24, *p* = .78, *η*^2^ = .003), or negative affect (*F*_2,143_ = .88, *p* = .42, *η*^2^ = .012). There was a significant effect of time (*F*_1.9,263.50_ = 3.40, *p* = .038, *η*^2^ = .02) on EDA, EE positive subscale (*F*_2.41,344.34_ = 74.32, *p* <.001, *η*^2^ = .34), and EE negative subscale (*F*_2.54,362.94_ = 38.96, *p* <.001, *η*^2^ = .21). Participants across conditions reported higher EDA, greater negative mood, and lower positive mood during the conversation task compared to at baseline (see Tables 3 and 4 for pairwise comparisons between all repeated measures). There were no main effects of condition (*F*_2,144_ = .10, *p* = .90, *η*^2^ = .001) or time (*F*_1,144_ = 1.24, *p* = .268, *η*^2^ = .009), nor was there a significant condition by time interaction (*F*_2,144_ = .78, *p* = .46, *η*^2^ = .01), on the anticipatory and peak SUDS. Controlling for DASS-DEP or SPIN scores had no impact on any findings (see Supplemental Materials: Supplementary Appendix A, Table A2).

**Table 4.** Pairwise comparisons between repeated measures.

	EDA				EEPOS				EENEG			
	*Mdiff*	*SE*	*p*	*g*	*Mdiff*	*SE*	*P*	*g*	*Mdiff*	*SE*	*p*	*g*
Baseline–Anticipation					6.47	.68	<.001	.41	−2.34	.76	.002	.21
Baseline–Conversation	−.09	.04	.02	.22	11.79	1.10	<.001	.74	−3.32	1.07	.002	.29
Baseline–Recovery	−.07	.04	.09	.16	11.64	.89	<.001	.72	4.94	.83	<.001	.46
Anticipation–Conversation					5.05	1.02	<.001	.30	-.98	.78	.21	.09
Anticipation–Recovery					4.90	.89	<.001	.28	7.28	.74	<.001	.71
Conversation–Recovery	.03	.03	.42	.04	−.14	.81	.858	.02	8.26	.79	<.001	.79

*Note:* EDA = electrodermal activity, EEPOS = emotional experience positive subscale. EENEG = emotional experience, negative subscale.

For the memory task (Table 3), there were no significant differences between conditions on the number of hits (*F*_2,139_ = 1.44, *p* = .24, *η*^2^ = .02), misses (*F*_2,139_ = .80, *p* = .45, *η*^2^ = .02), recall of standardized information (*F*_2,140_ = .03, *p* = .97, *η*^2^ = .003), cued recall (*F*_2,144_ = .44, *p* = .64, *η*^2^ = .006), or recognition (*F*_2,144_ = .59, *p* = .94, *η*^2^ = .008).

Given that both attrition and our manipulation check method resulted in uneven cell sizes, we re-ran our main analyses on an exploratory basis with all participants included to assess the potential impact of uneven attrition. There were no main effects of condition on EDA (*F*_4_,_295_ = .77, *p* = .54), positive affect (*F*_6_,_284_ = 1.73, *p* = .13), negative affect (*F*_6_,_292_ = .99, *p* = .42), recall of standardized information (*F*_2,154_= .402, *p* = .67), cued recall (*F*_2,152_ = .682, *p* = .51), or recognition (*F*_*2,154*_ = .254, *p* = .78).

Additionally, we performed exploratory analyses excluding participants who reported a past psychological disorder diagnosis. We found no differences between conditions on EDA (*F*_*3.65*_*,*_*165.78*_ = 2.34, *p* = .064), positive affect (*F*_4.67,268.34_ = 1.78, *p* = .122), negative affect (*F*_4.60,264.75_ = .320, *p* = .882), recall of standardized information (*F*_2,115_ = .097., *p* = .908), cued recall (*F*_2,228_ = .110, *p* = .896), or recognition (*F*_*2,118*_ = .128, *p* = .88).

### Interpersonal consequences

There were significant differences between conditions on the proportion of positive expressions (*F*_2,140_ = 37.27, *p* <.001, *η*^2^ = .35) and neutral expressions (*F*_2,140_ = 41.32, *p* <.001, *η*^2^ = .37), across conditions, during the conversation task, providing further evidence that our manipulation was effective. Participants in the expressive suppression condition displayed significantly more neutral facial expressions than those in the control (*Mdifference =*.21, *p* <.001, *g* =1.09) and dissonance (*Mdifference =*.35, *p* <.001, *g* =1.76) conditions. Participants in the expressive dissonance condition showed significantly more positive facial expressions than those in the control (*Mdifference =*.16, *p* <.001, *g* =.72) and suppression conditions (*Mdifference =*.34, *p* <.001, *g* = 1.76). Participants in the control condition exhibited significantly more positive expressions (*Mdifference =*.19, *p *<.001, *g* = 1.04) and significantly fewer neutral expressions (*Mdifference =* −.21, *p* <.001, *g* = 1.13) than the expressive suppression condition. There were no differences between conditions on seconds of unresponsiveness (*F*_2,140_ = .54, *p* = .58, *η*^2^ = .008).

Participants engaging in dissonance were rated as significantly more friendly, likeable, warm, and approachable than those in the suppression and control conditions. They were also rated as significantly more easy-doing thatn those in the suppression, but not control condition. Participants in the suppression condition were rated as significantly less friendly, likeable, warm, approachable, and easy-going than those in the dissonance and control conditions (see Table 5). We also conducted exploratory analyses excluding participants who reported a past psychological disorder diagnosis. Our results were virtually identical to our planned analyses (see Supplemental Materials: Supplementary Appendix B).

**Table 5.** Pairwise comparisons between conditions on interpersonal qualities.

	Dissonance–suppression				Dissonance–control				Control–suppression			
Interpersonal qualities	*Mdiff*	*SE*	*p*	*G*	*Mdiff*	*SE*	*p*	*g*	*Mdiff*	*SE*	*p*	*g*
Friendly	1.47	.17	.000	1.80	.54	.18	.002	.64	.92	.17	.000	1.05
Likeable	.97	.18	.000	1.09	.57	.19	.003	.61	.40	.18	.03	.44
Warmth	1.44	.22	.000	1.39	.52	.22	.02	.45	.92	.22	.000	.90
Approachable	1.11	.22	.03	1.03	.48	.22	.03	.45	.64	.22	.004	.60
Easy-going	.71	.22	.001	.67	.26	.22	.24	.24	.45	.22	.04	.42

## Discussion

We examined whether using expressive dissonance leads to better *intrapersonal* (i.e. psychophysiology, memory accuracy, self-reported affect – positive and negative mood and anxiety) and *interpersonal* (i.e. interpersonal qualities) outcomes than expressive suppression, among women engaging in a conversation task with an unacquainted male confederate. Although the conversation task was distressing for all participants (as indicated by EDA and self-report questionnaires), there was no advantage of one strategy over another or over control for intrapersonal outcomes. There were, however, important differences on interpersonal outcomes. People who used expressive dissonance (i.e. smiling) were rated more positively, and people who used expressive suppression (i.e. poker face) were rated more negatively, on multiple interpersonal qualities, relative to those in the control condition.

Our findings suggest that although intentionally showing outward positive emotional expression, even when one is feeling anxious, may not lead to positive internal outcomes, it may lead to temporary positive social outcomes. Moreover, the positive and negative interpersonal consequences of using expressive dissonance and expressive suppression, respectively, are likely attributable to impressions by *others* rather than to (in)effective intrapersonal ER. Our findings contribute to the relationship development literature, which suggests that positive emotion expression communicates affiliation, leading one to be perceived as approachable and friendly (Harker & Kelner, 2001).

According to Zaki and Williams’ (2013) model, interpersonal ER involves an (1) extrinsic interpersonal factor that regulates the perceiver and/or (2) intrinsic interpersonal goal to manage one’s emotions. Expressive suppression or dissonance did not help regulate one’s emotions, given our observed lack of effect on intrapersonal factors. It is possible that smiling or neutral expressions impacted the emotions felt by the observer (i.e. the video coders), thereby impacting their judgements of the participant. Notably, Nevin (2017) contends that intention to influence another person’s emotion is necessary for a behaviour to be considered interpersonal ER. Although we asked participants to use a specific ER strategy, we gave them no indication that they should do so to influence the confederate’s emotions. We did suggest they acquaint themselves to that person similar to what they would usually do, which may have activated interpersonal ER intentions. Because we used a confederate to increase internal validity and allow for experimental control for the memory task, we are unable to explore effects on the conversation partner. Future research focused on interpersonal ER intentions and conversation partners’ satisfaction and beliefs around relationship formation may be particularly fruitful.

Additionally, video coders may have rated participants more favourably when expressing positive emotion due to the participant’s situation-congruent affect. A getting acquainted/friendship-forming task is generally a ‘positive’ social situation. Although the task was distressing for the regulator (as demonstrated by increased EDA and negative affect and reduced positive affect), expressing positive emotions in a situation that is *considered* positive by the observer (who again, was unaware of the study goals or manipulation) may lead to positive social judgement. Perhaps expressing emotions considered *incongruent* to the situation (i.e. showing a neutral expression during a getting acquainted task) leads to negative ratings on social variables. This suggestion is consistent with research findings that participants who displayed affect incongruent with the stimuli (e.g. positive affect in response to negative images) or neutral affect (i.e. no response to the stimuli), were judged more negatively than those displaying congruent affect (Szczurek et al., 2012). Additionally, women may be judged particularly harshly for displaying incongruent affect. Brown et al. (2015) found that women were rated significantly more negatively than men when showing incongruent affect toward images. Indeed, a recent meta-analysis found poorer social outcomes for women in relation to emotion suppression, but no gender differences in relation to emotion expression, suggesting that affect incongruence may be particularly harmful to women’s social well-being (Chervonsky & Hunt, 2017).

Because we recruited an unselected sample of women, we are unsure whether gender differences and gender norms impacted the results. According to Hess and Hareli (2015), gendered display rules outline socially appropriate displays of facial expression, which lead to gender differences between the ‘appropriate’ expressions for men and women. Their findings suggest that women and men both believe women are more expressive than men and that women smile more than men (Adams et al., 2015; Hess & Hareli, 2015). Nonsmiling women are judged as experiencing more negative affect than nonsmiling men and perceived more unfavourably than smiling women (Deutsch et al., 1987). It is therefore unclear whether our findings reflect a particular bias toward unsmiling women (e.g. as unfriendly) or a universal impact of smiling during an interpersonal interaction. It is crucial to expand on our work to include men to examine whether response-focused ER strategies carry the same interpersonal consequences across genders. Moreover, although our participants reported diverse racial/ethnic backgrounds and sexual orientations, we did not ask about disabilities other than a diagnosed mood or anxiety disorder. As such, we cannot generalize our findings beyond diverse undergraduate women.

Our lack of between condition intrapersonal differences aligns with findings from our previous study (Bahl & Ouimet, 2022), but contrasts with a large body of literature suggesting that expressive suppression is always maladaptive. Previously, we found that participants used multiple ER strategies during the stressful task, regardless of condition (Bahl & Ouimet, 2022). In our current study, 63% of participants in the expressive suppression condition, 77% of the expressive dissonance condition and 73% of the control condition reported changing the interpretation of the task to make it appear less distressing (i.e. cognitive reappraisal; see Supplemental Materials: Supplementary Appendix A, Table A1).

We wonder if the lack of intrapersonal differences is attributable to the coactivation of multiple ER strategies, thereby reducing the effect of the manipulation. This possibility is consistent with Gross and Thompson (2007), who suggest that people may use multiple strategies simultaneously. It also raises important questions about the limitations inherent in manipulating single ER strategies in experimental research. First, it almost certainly reduces ecological validity. Second, it likely artificially increases or decreases effect sizes, depending on which other strategies people use, how they are measured and the context in which participants are asked to use them (see Bahl and Ouimet (2022) for more detail).

Nonetheless, our findings suggest that specific ER strategy use may impair interpersonal (i.e. relationship building) more so than intrapersonal functioning (i.e. affecting oneself). It appears that even if people use additional strategies (i.e. cognitive reappraisal, a relatively healthy ER strategy) to manage distress and anxiety in social situations, the specific expressive strategy presented holds interpersonal consequences. It is vital that future research focus on measuring the additive and interactive effects of multiple emotion regulation strategy use on intrapersonal and interpersonal outcomes*.*

Additionally, it is possible that to adequately engage in expressive dissonance, one may have to first suppress their felt emotion and then display or exaggerate the required/assigned emotion. Indeed, in our manipulation check, over half of participants in the expressive dissonance condition indicated that they maintained a neutral expression somewhat or very often during the conversation task. It is therefore possible that our lack of intrapersonal findings is attributable to an overlap of strategies between the expressive suppression and expressive dissonance group. Notably, however, neither of the experimental conditions was different from the control condition, suggesting that neither expressive strategy is advantageous or disadvantageous in managing felt emotions compared to simply expressing felt emotions as they occurred.

In conclusion, we examined the interpersonal and intrapersonal consequences of using expressive dissonance or expressive suppression during a conversation task with an unacquainted confederate partner. Although there were no differences in intrapersonal consequences (changes in sympathetic nervous system, memory accuracy or self-reported affect), we found that people who engaged in expressive dissonance were rated more positively, and people who used expressive suppression were rated more negatively in interpersonal characteristics important for a positive conversation. Our findings suggest that if one’s objective is to build social bonds and likeability, then expressive dissonance (i.e. smiling) when feeling anxious may facilitate this goal.

## Supplemental Material

sj-pdf-1-spr-10.1177_02654075221077233 – Supplemental Material for Smiling won’t make you feel better, but it might make people like you more: Interpersonal and intrapersonal consequences of response-focused emotion regulation strategies Click here for additional data file. Supplemental Material, sj-pdf-1-spr-10.1177_02654075221077233 for Smiling won’t make you feel better, but it might make people like you more: Interpersonal and intrapersonal consequences of response-focused emotion regulation strategies by Nancy Bahl and Allison J Ouimet in Journal of Social and Personal Relationships

## Notes

1 The original dataset can be found at: osf.io/sg8fa.

2 Given the similarity of measures and experimental manipulation used in this study and Bahl and Ouimet (2022), there is considerable overlap in the text of the Methods sections of these two articles. The two experiments differ in the behavioural task assigned and the outcomes measured. In Bahl and Ouimet (2022), participants responded to negative emotion-eliciting images while we measured intrapersonal outcomes only; in the current study participants completed a conversation task with a confederate, which enabled us to also measure interpersonal outcomes.

3 https://journals.sagepub.com/doi/suppl/10.1177/02654075221077233 Please see Supplementary Appendix B for a description of the G*Power Analysis.

4 Participants could select as many descriptors as were relevant; percentages add up to more than 100%.
