# Supplementary material for: Smiling won’t make you feel better, but it might make people like you more: Interpersonal and intrapersonal consequences of response-focused emotion regulation strategies
Source: J Soc Pers Relat. 2022 Feb 28;39(7):2262–84. doi: 10.1177/02654075221077233 (PMC9210114; doi:10.1177/02654075221077233)
Supplement: sj-pdf-1-spr-10.1177_02654075221077233 – Supplemental Material for Smiling won’t make you feel better, but it might make people like you more: Interpersonal and intrapersonal consequences of response-focused emotion regulation strategies [file sj-pdf-1-spr-10.1177_02654075221077233.pdf]

Appendix A

Table A.1

Frequencies and Percentages for Questions (#) in the Manipulation Check Questionnaire

	Expressive Suppression					Expressive Dissonance					Control				
	0 (%)	1 (%)	2 (%)	3 (%)	4 (%)	0 (%)	1 (%)	2 (%)	3 (%)	4 (%)	0 (%)	1 (%)	2 (%)	3 (%)	4 (%)
#1	1 (1.9)	2 (3.8)	23 (43.4)	27 (50.9)	0	0	17 (36.2)	25 (53.2)	5 (10.6)	0	0	13 (27.7)	18 (38.3)	15 (31.9)	1 (2.1)
#2	1 (1.9)	18 (34.0)	26 (49.1)	8 (15.1)	0	0	0	7 (14.9)	36 (76.6)	4 (8.5)	1 (2.1)	3 (6.4)	17 (36.2)	22 (46.8)	4 (8.5)
#3	11 (20.8)	11 (20.8)	17 (36.2)	14 (26.4)	0	5 (10.6)	6 (12.8)	21 (44.7)	11 (23.4)	4 (8.5)	3 (6.4)	10 (21.3)	13 (27.7)	16 (34.0)	5 (10.6)
#4	9 (17.0)	11 (20.8)	19 (35.8)	10 (18.9)	4 (7.5)	6 (12.8)	7 (14.9)	15 (31.9)	15 (31.9)	4 (8.5)	5 (10.6)	14 (29.8)	13 (27.7)	7 (14.9)	8 (17.0)

Note: Responses: 0 = Not at all, 1 = Rarely, 2 = Somewhat, 3 = Very Often, 4= Always; Items: #1 = "…maintained a neutral expression?", #2 "…maintained a smile?", #3 = "…changed the interpretation of the conversation to make it less distressing?", #4 = "…find the conversation anxiety-provoking?"

Table A.2

Main Analyses with DASS-DEP and SPIN as Covariates

Predictor	Sum of Squares	*df*	Mean Square	*F*	*p*	η^2
EDA	.56	1.93	.29	3.06	.05	.02
EDA*SPIN	.19	1.93	.10	1.06	.34	.008
EDA*Condition	.27	3.86	.07	.73	.57	.01
Error	24.94	262.14	.09			
EDA	.53	1.90	.28	2.92	.06	.02
EDA*DASSDEP	.09	1.90	.05	.51	.59	.004
EDA*Condition	.33	3.81	.09	.91	.45	.01
Error	24.37	256.87	.09			
EEPOS	2401.63	2.39	1002.79	13.49	.000	.09
EEPOS*SPIN	403.79	2.39	168.60	2.27	.09	.02
EEPOS*Condition	444.63	4.79	92.83	1.25	.29	.02
Error	25111.17	337.69	74.36			
EEPOS	6971.89	2.45	2843.41	38.66	.000	.22
EEPOS*DASSDEP	189.03	2.45	77.09	1.05	.36	.007
EEPOS*Condition	380.56	4.90	77.60	1.05	.38	.02
Error	25245.08	343.27	73.54			
EENEG	555.91	2.53	219.47	3.76	.02	.03
EENEG *SPIN	906.95	2.53	358.06	6.15	.001	.04
EENEG*Condition	267.15	5.07	52.74	.91	.48	.01
Error	20796.97	357.15	58.23			
EENEG	2486.10	2.55	973.52	16.16	.000	.10
EENEG*DASSDEP	132.88	2.55	52.03	.86	.44	.006
EENEG*Condition	209.67	5.11	41.05	.68	.64	.01
Error	21542.67	357.52	60.26			

Note: DASS-DEP = Depression Anxiety Stress Scale, Depression Subscale, SPIN = Social Phobia Inventory, EDA = Electrodermal Activity, EEPOS1 = emotional experience positive subscale; EENEG = emotional experience, negative subscale

Appendix B

Exploratory Analyses

We also conducted exploratory analyses excluding participants who reported a past psychological disorder diagnosis. Similar to our planned analyses, there were significant differences between conditions on the proportion of positive expressions ($F_{2,115}$ =28.43, p <.001) and neutral expressions ($F_{2,115}$ =29.68, p <.001). Those in the expressive dissonance conditioned displayed more positive facial expressions than those in the suppression (*Mdifference*=.34, p <.001) and control condition (*Mdifference*=.17, p <.001). Those in the expression suppression condition showed significantly more neutral expressions than those in the control (*Mdifference*=.19, p <.001 or dissonance condition (*Mdifference*=.34, p <.001). Those in the control condition exhibited significantly more positive expressions (*Mdifference*=.18, p<.001) and significantly fewer neutral expressions (*Mdifference*= -.19, p<.001) than the expressive suppression condition. Additionally, there were no differences between conditions on seconds of unresponsiveness ($F_{2,115}$ =.49, p =.61). There were significant differences between condition on interpersonal qualities: friendly ($F_{2,115}$ =31.23, p <.001), likeable ($F_{2,115}$ =11.69, p <.001), warm ($F_{2,115}$ =21.38, p <.001), approachable ($F_{2,115}$ =12.46, p <.001), and easy-going ($F_{2,115}$ =5.2, p =.007). Participants engaging in dissonance were rated as significantly more friendly, likeable, warm, and easy-going, than those in the suppression and control conditions, and participants in the suppression condition were rated as significantly less friendly, likeable, warm, approachable, and easy-going than those in the dissonance and control conditions.

Appendix C

Check-in Questionnaire

0<br>Not at all	1<br>Rarely	2<br>Somewhat	3<br>Very Often	4<br>Always

1. How often did you show an expressionless or neutral expression throughout the conversation task? _____

2. How often did you show happiness or smile throughout the conversation task? _____

3. How often did you try to change your interpretation of the conversation task to make it less distressing? _____

4. Did you find the conversation task anxiety provoking?_____

5. Did you do anything to make the conversation task less distressing/anxiety-provoking? Please explain.
